# Optimizing Valve Selection in Valve-in-Valve Transcatheter Aortic Valve Replacement: A Case Study on Addressing Patient-Prosthesis Mismatch and Early Structural Valve Deterioration in a Morbidly Obese Patient

**DOI:** 10.7759/cureus.53191

**Published:** 2024-01-29

**Authors:** Victor H Molina-Lopez, Ismael Ortiz-Cartagena, Josue Mercado-Crespo, Miguel A Campos-Esteve

**Affiliations:** 1 Cardiology, Veterans Affairs Medical Center, San Juan, PRI; 2 Interventional Cardiology, Hospital Pavia, San Juan, PRI

**Keywords:** transcatheter heart valve, structural valve deterioration, valve-in-valve, transcatheter aortic valve replacement, patient-prosthesis mismatch

## Abstract

Transcatheter aortic valve replacement (TAVR) has increasingly become a fundamental approach for treating aortic valve stenosis (AVS), especially in high surgical risk patients. This case study underscores the criticality of meticulous procedural planning and precise valve selection in patients with severe AVS compounded by obesity. We report a case of a patient who, after receiving a 26 mm Edwards Sapiens 3 valve, presented with worsening exertional dyspnea and a declining indexed effective orifice area (EOAi). This deterioration indicated early structural valve deterioration (SVD), presumably due to patient-prosthesis mismatch (PPM). A subsequent valve-in-valve (ViV) TAVR using a 29 mm Medtronic Evolut Fx valve was successfully executed, leading to a notable improvement in EOAi. This case study emphasizes the complexities inherent in valve choice and sizing in TAVR, particularly highlighting the impact of PPM on obese patients and its potential to precipitate early SVD. The report further explores the emerging strategies in addressing TAVR valve dysfunctions via ViV interventions, shedding light on the nuanced and dynamic nature of TAVR management in obese patients. It advocates for tailored treatment strategies in managing such intricate cases, demonstrating the evolving landscape of TAVR procedures.

## Introduction

Transcatheter aortic valve replacement (TAVR) represents a paradigm shift in treating aortic valve stenosis (AVS), particularly for patients at elevated risk from conventional surgical aortic valve replacement (SAVR). This shift is mainly attributable to advancements in bioprosthetic valve designs and the refinement of catheter-based implantation techniques. These advancements have broadened TAVR's applicability, including for patients with complex comorbidities, thereby solidifying its essential role in contemporary cardiological practice [1].

This case report presents a complex instance of TAVR in a 75-year-old male patient with severe AVS, further complicated by obesity (Body Mass Index of 45 kg/m²) and early onset structural valve deterioration (SVD) due to patient-prosthesis mismatch (PPM). Initially treated with a 26 mm Edwards Sapien 3 valve (Edwards Lifesciences, Irvine, CA, USA), the patient later showed a significant decline in indexed effective orifice area (EOAi) and exacerbated clinical symptoms. This necessitated a challenging valve-in-valve (ViV) TAVR re-intervention using a 29 mm Evolut Fx valve (Medtronic, Minneapolis, MN, USA). This report critically examines the complex considerations in valve selection and sizing in TAVR, focusing on managing PPM in obese patients. Additionally, it explores the evolving strategies for addressing TAVR-related valve dysfunctions and the strategic planning of ViV procedures, thereby highlighting the intricate, dynamic nature of TAVR in patients with unique anatomical and comorbidity challenges.

## Case presentation

We present the case of a 75-year-old male who was evaluated for transcatheter heart valve (THV) stenosis and progressive dyspnea on exertion. He had TAVR for severe AVS two years prior with an Edwards Sapiens 3 balloon-expandable THV. The patient's medical history is notable for type 2 diabetes mellitus, hypertension, hyperlipidemia, coronary artery disease with prior percutaneous coronary intervention, permanent atrial fibrillation with a CHA2DS2-VASc score of 7 on rivaroxaban, chronic diastolic heart failure, morbid obesity, chronic obstructive pulmonary disease (COPD), obstructive sleep apnea, and chronic venous insufficiency.

The patient's initial AVS diagnosis was classified as stage D1, with pre-procedural transthoracic echocardiogram (TTE) findings consistent with a heavily calcified tri-leaflet aortic valve with a valve area (AVA) of 0.69 cm2 by the continuity equation (with an AVA indexed by body surface area (BSA) (AVAindex [AVAi]) of 0.26 cm²/m²), mean gradient of 42 mmHg, and peak jet velocity (Vmax) of 4.3 m/sec. The aortic root was normal in size. Left ventricular ejection fraction (LVEF) was preserved at 60%, with diastolic dysfunction evident in the setting of atrial fibrillation, mild left ventricular hypertrophy, mild mitral annular calcification, bi-atrial enlargement, with mild-to-moderate tricuspid regurgitation, and mild pulmonary artery hypertension. Initial evaluation for cardiac amyloidosis was unremarkable. The patient had symptoms consistent with a functional classification Class III according to the New York Heart Association (NYHA) Heart Failure Criteria. The patient's Body Mass Index (BMI) was 45 kg/m² with a BSA of 2.6 m². He had multiple admissions for treatment of heart failure, deemed secondary to the severe prosthetic aortic valve stenosis. The Society of Thoracic Surgeons (STS) predicted surgical mortality risk was 10% with a moderate frailty index, guiding the decision towards TAVR by Heart Team approach and patient preference. Electrocardiogram (ECG) pre-implant was remarkable for atrial fibrillation with adequate ventricular response and no evidence of bundle branch block. Hemoglobin was 15.1 g/dL, blood urea nitrogen (BUN) 11 mg/dL, and creatinine 0.8 mg/dL. Coronary angiography was unremarkable for obstructive coronary artery disease (CAD).

**Figure 1:**
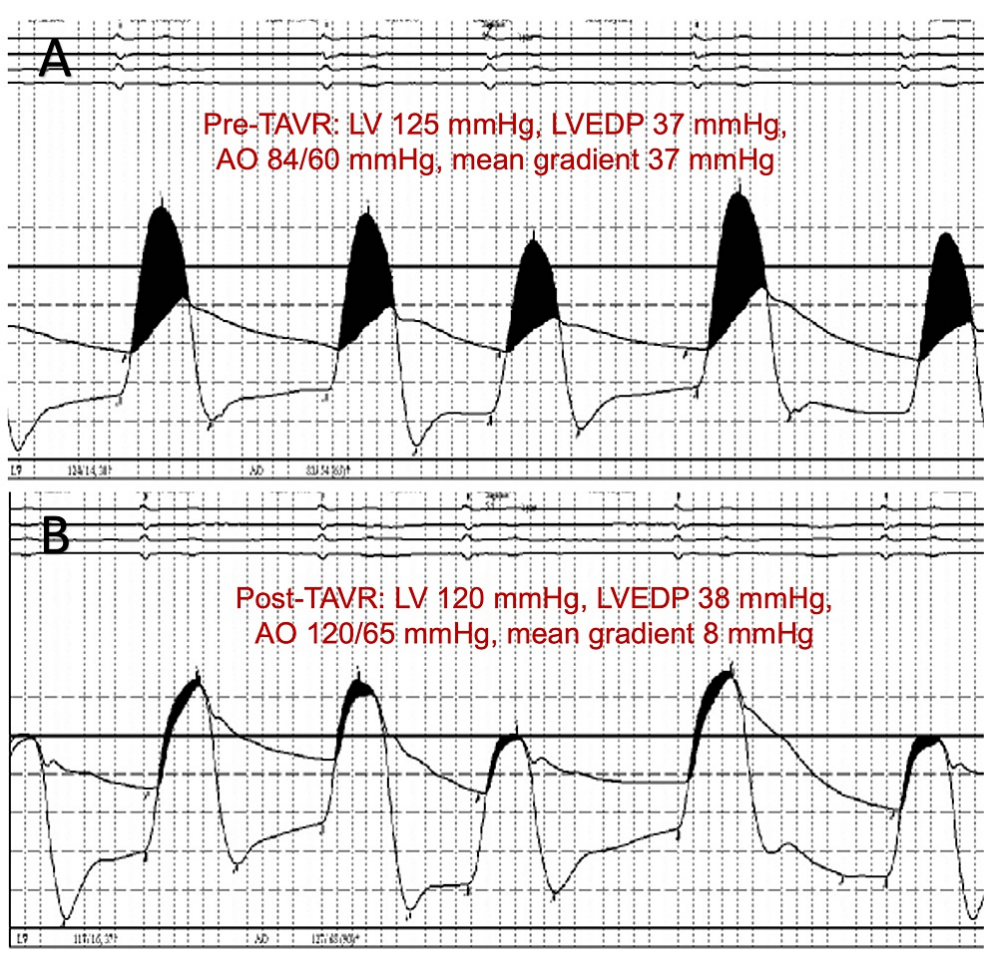
Invasive hemodynamics during index S3 valve implant. (A) Pre-TAVR and (B) post-TAVR invasive hemodynamics demonstrating successful reduction of the mean gradient after implantation of the index S3. TAVR: Transaortic Valve Replacement; LV: Left ventricular pressure; LVEDP: Left ventricular end-diastolic pressure; Ao: aortic pressure; S3: Edwards Sapiens 3 pericardial tissue valve

Computerized Tomographic Angiography (CTA) analysis with TAVR protocol was employed for pre-procedural planning. Coronary ostia measurements were 16.4 mm for the left coronary ostia and 13.5 mm for the right coronary ostia. The mean diameter at the sinus of Valsalva was 32 mm. The annulus had an area of 475 mm2, with a mean diameter of 24.6 mm. The aortic root angle was 62 degrees. The index valve implant technique involved a femoral arterial access approach with a three-cusp co-planar view for implant deployment under fluoroscopy guidance. A pre-deployment balloon aortic valvuloplasty was conducted with a 22 mm True Dilation Balloon (Bard, Murry Hill, NJ, USA), followed by the delivery and implant of a balloon expandable 26 mm Edwards Sapiens 3 pericardial tissue valve at nominal filling pressure. Hemodynamic parameters were closely monitored, with pre-TAVR and post-TAVR measurements demonstrating a mean gradient of 8 mmHg (Figure 1A-1B). Post-deployment transitory left bundle branch block occurred, which precluded further post-deployment oversizing or valve optimization. The immediate post-procedural course was otherwise unremarkable for complications. Anticoagulation with rivaroxaban was promptly resumed after the procedure.

Post-implant TTE revealed an EOAi of 0.52 cm²/m². Follow-up TTE studies over the next two years revealed progressive worsening of the EOAi concerning for early SVD as a consequence of PPM (Table 1).

**Table 1:** Echocardiographic data after implant of index S3 prosthetic heart valve after the TAVR, at one-year follow-up, at two-year follow-up, and after the Evolut-in-S3 ViV TAVR. PrAV: Prosthetic aortic valve; TTE: Transthoracic echocardiogram; TAVR: Transaortic valve replacement; ViV: Valve-in-valve; BMI: Body mass index; BSA: Body surface area; Vmax: Max prosthetic aortic valve Doppler velocity; DVI: Doppler velocity index; EOA: Effective orifice area; EOAi: Indexed effective orifice area; S3: Edwards Sapiens 3 balloon expandable valve; Evolut: Medtronic Evolut FX self-expanding valve

PrAV TTE Metrics	Post-TAVR	1-year follow-up	2-year follow-up	Post-ViV TAVR
BMI (kg/m²)	45	45	45	45
BSA (m²)	2.6	2.6	2.6	2.6
Vmax (m/sec)	1.75	2.70	3.75	2.00
Mean gradient (mmHg)	8	18	32	6
DVI	0.43	0.34	0.31	0.67
EOA (cm²)	1.36	1.06	0.88	2.11
EOAi (cm²/m²)	0.52	0.41	0.35	0.81

Over the next two years after the index valve implant, the patient's dyspnea progressively worsened, escalating from NYHA functional class II to IV, along with exertional angina. Coronary angiography revealed no significant obstructive CAD. Even with strict adherence to maximally tolerated guideline-directed medical therapy (GDMT), the symptoms persisted, and readmissions increased in frequency again. Consequently, after Heart Team re-discussion, the patient was referred for a ViV TAVR to address severe early SVD and stenosis of the Sapien 3 valve.

CTA analysis with TAVR protocol was employed for ViV TAVR pre-procedural planning. The etiology of early SVD was deemed secondary to prosthetic valve calcific deterioration, leading to prosthetic valve stenosis. The possibility of subacute valve thrombosis after implanting the index S3 valve was considered; however, no evidence of thrombosis was present on follow-up TTE, and CT analysis failed to show evidence of leaflet hypoattenuation. CT-based sizing for the index Sapien 3 was performed to assess the best ViV strategy. Our assessment focused on several key aspects. We evaluated the coronary risk plane (CRP) for potential coronary obstruction and interference of coronary access after redo-TAVR. In the neoskirt plane (NSP), we assessed new valve placement in relation to the existing Sapien3 valve, particularly for S3-in-S3 and Evolut-in-S3 scenarios. The valve-to-aorta (VTA) distance was meticulously measured to ensure spatial compatibility, which is especially important in S3-in-S3 configurations. Finally, we evaluated the possibility of leaflet modification according to coronary ostium eccentricity (COE), focusing on whether the angle between the coronary ostium and the center of the S3 leaflet fell within the range of 0° to 20°. However, in this instance, such modification was not required. The index Sapien 3 implant depth was 3-4 mm. The mean annulus diameter of the new aortic annulus with the S3 valve was 24.5 mm, with a mean sinus of Valsalva diameter of 32.6 mm. A 29 mm Evolut Fx self-expanding valve was selected as the most suitable ViV strategy and offered an 18% oversizing percentage.

**Figure 2:**
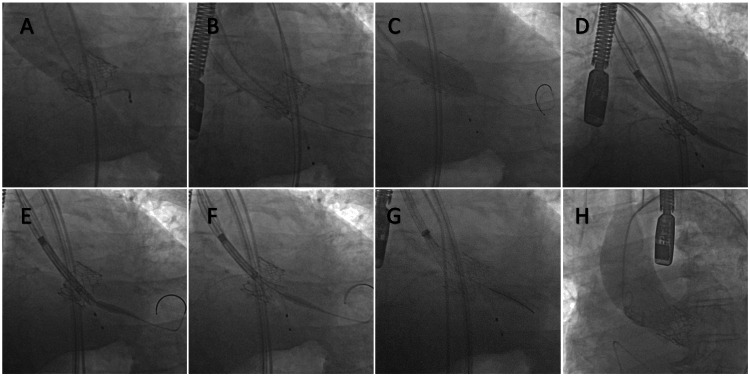
TEE- and fluoroscopy-guided Evolut-in-S3 ViV TAVR. TEE: Transesophageal echocardiography; TAVR: Transaortic valve replacement; ViV: Valve-in-valve; S3: Edwards Sapiens 3 pericardial tissue valve; Evolut: Medtronic Evolut FX self-expanding valve

The ViV procedure was performed via femoral arterial access for an Evolut-in-S3 ViV TAVR implant strategy. Pre-dilation valvuloplasty was performed using a 20 mm True Dilation Balloon under standard rapid pacing to open the heavily calcified 26 mm S3 valve and allow for equipment delivery (Figure 2C). Subsequently, the 29 mm Evolut FX was introduced, the position marked with the prior TAVR valve border, and was subsequently deployed using the standard slow deployment technique (Figure 2D-2H). Excellent expansion and coaxial valve deployment were achieved. Repeat hemodynamic measurements revealed a mean peak gradient of 2 mmHg. Transesophageal echocardiography (TEE) revealed no paravalvular leak with adequate valve function. Post-procedural TTE revealed an improved mean gradient of 6 mmHg, EOA of 2.11 cm², and EOAi of 0.82 cm²/m² (Table 1). The procedure was complicated by an irreversible complete atrioventricular block after deployment of the self-expanding valve, leading to permanent pacemaker implantation. Otherwise, the patient had an excellent post-procedural course with marked improvement of his dyspnea and exertional angina resolution, with a residual functional class improvement to NYHA I. Anticoagulation was resumed for life-long anticoagulation for his permanent atrial fibrillation with no clinically significant bleeding events.

## Discussion

In this case, we examined a 75-year-old male patient who underwent a ViV TAVR to address severe prosthetic AVS attributed to early SVD. This SVD was associated with PPM, likely influenced by the patient's severe obesity (BMI of 45 kg/m²) and cardiac output requirements. While the initial placement of a 26 mm Edwards Sapien S3 valve was technically successful, subsequent TTE revealed suboptimal hemodynamics for his body habitus, evidenced by an EOAi of 0.52 cm²/m². Within two years, the transvalvular hemodynamics worsened and progressed to early SVD, characterized by calcification and stenosis of the aortic valve prosthesis. These complications indicate a possible oversight in the original valve sizing or balloon expansion approach, potentially failing to consider the patient's high BSA and BMI. 

Evaluating valve dysfunction post-TAVR necessitates immediate post-implantation hemodynamic assessments and ongoing follow-up examinations. The primary evaluation after TAVR focuses on assessing the valve's placement, the uniformity of the stent shape, the structural integrity and motion of the leaflets, and comprehensive hemodynamic analysis. This hemodynamic evaluation should encompass flow-dependent factors, such as the mean gradient, and flow-independent measures, such as the EOA. When there is a discrepancy between these measurements, the Doppler velocity index (DVI) is determined. An abnormal DVI suggests potential prosthetic valve malfunction, while a normal DVI implies inherent prosthetic valve adequacy, allowing for indexed EOA utilization to elucidate the cause of initial measurement discrepancies. If the EOAi is reduced despite a normal DVI, PPM is a probable cause, indicating a mismatch between the valve's functionality and the patient's cardiac output requirements [1]. PPM in TAVR occurs when the EOA of the prosthetic valve is insufficient relative to the patient's body size. In both new and ViV TAVR procedures, the assessment of PPM risk is based on the EOAi from the selected second TAVR device [1,2]. For patients with BMI <30 kg/m², classifications are: no PPM at EOAi >0.85 cm²/m², moderate at 0.66-0.85 cm²/m², severe ≤0.65 cm²/m². For BMI ≥30 kg/m², they are: no PPM at EOAi >0.70 cm²/m², moderate at 0.56-0.70 cm²/m², severe ≤0.55 cm²/m² [1-6].

PPM is associated with increased mortality, heart failure rehospitalization, and early valve deterioration [1-8]. Moreover, even moderate PPM has been shown to increase late mortality in patients with left ventricular dysfunction, but its impact on those with preserved left ventricular function is generally more favorable [6]. Factors such as age, BMI, and valve choice influence PPM risk, and prevention strategies include careful patient and valve selection [9-11]. Elevated BSA and obesity significantly influence valve sizing. PPM in patients with high BSA or obesity can result in increased prosthetic gradients, worse hemodynamic function, and reduced survival [1]. 

PPM following aortic valve replacement has several physiological consequences, particularly concerning early SVD. PPM is related to early stenosis-type SVD, which can be preventable with proper valve selection and readily identified through echocardiography and other imaging modalities [12,13]. In addition, incompetence-type SVD, characterized as time-dependent, nonspecific wear damage, can also be associated with PPM [12]. Early SVD in patients with PPM often exhibit decreased regression of left ventricular hypertrophy, reduced coronary flow reserve, and an increased incidence of congestive heart failure, all of which contribute to diminished functional capacity and increased risk of early and late mortality [14,15]. PPM negatively impacts prognosis after valve replacement, increasing all-cause and cardiac mortality, and showing a close relationship with SVD in biological prostheses [16,17].

The ViV procedure has emerged as a viable therapeutic strategy for addressing failed TAVR valves. A key technical aspect of ViV procedures, particularly when addressing a previously implanted Edwards valve, involves balloon dilation. This is a critical step to secure the proper seating and expansion of the new valve within the existing structure. The choice between self-expanding and balloon-expandable valves like Edwards' Sapien depends on patient-specific factors, including anatomy and BSA [2]. Both valve types have shown comparable clinical outcomes and mortality rates in TAVR procedures [18]. As a general rule, balloon expandable prosthetic valves tend to "remodel" the annulus on implant [19]; however, in self-expanding aortic valve prosthesis, the annulus tends to remodel the prosthetic valve [20].

ViV TAVR procedures demonstrate high procedural success rates and are associated with lower risks of operative mortality and postoperative complications compared to redo-AVR in failed aortic valve bioprosthesis [21]. Meticulous pre-procedural planning and intra-procedural imaging are essential to minimize risks such as coronary obstruction, valve malposition, and conduction abnormalities. Long-term follow-up strategies include regular echocardiographic assessment to monitor valve function and vigilant surveillance for signs of structural valve deterioration, endocarditis, and thrombosis [22,23]. While ViV-TAVR shows better short-term outcomes than redo SAVR, major cardiovascular outcomes appear unchanged during long-term follow-up, emphasizing the need for ongoing patient evaluation and management [24]. 

In TAVR procedures for obese patients, valve sizing, particularly oversizing, plays a crucial role in outcomes. Oversizing TAVR valves in this demographic may prevent paravalvular leak (PVL) and mitigate PPM [25]. Optimal sizing in self-expanding TAVR valves lies between 10-20% oversizing [26]. However, oversizing comes with risks, such as annular rupture or valve thrombosis [27,28]. Oversizing by over 20% has been associated with an increased risk of hypoattenuating leaflet thickening [28]. Larger ascending aortic dimensions and smaller degrees of oversizing are significant predictors of unsuccessful device implantation in self-expanding TAVR valves [29]. Excessive perimeter oversizing and implantation depths greater than 6 mm should be avoided to reduce the risk of complications like permanent pacemaker implantation [30]. Additionally, moderate valve oversizing in self-expanding transcatheter aortic valves increases leaflet bending stress during opening and closing, which could impact long-term durability [31]. Irrespective of whether the valve is oversized or undersized, and regardless of the valve model used, a borderline annulus is associated with a notably higher transvalvular gradient and greater incidence of PVL in comparison to cases with a non-borderline annulus during TAVR [32].

The evolution of prosthesis iterations, including both self-expanding and balloon-expandable transcatheter heart valves, has been shown to reduce the incidence of PPM after TAVR [33]. However, comparisons between self-expandable CoreValve bioprostheses and balloon-expandable Edwards Sapien bioprostheses have revealed that while the CoreValve shows better hemodynamic performance, the incidence of PVL is higher with this prosthesis compared to the Edwards Sapien valve [34]. Additionally, next-generation self-expanding valves (ACURATE neo) have demonstrated better hemodynamic performance and lower rates of severe PPM compared to balloon-expandable transcatheter aortic valves in patients with small aortic annuli [35]. Furthermore, balloon-expandable Sapien valves have been associated with shorter fluoroscopy times and lower aortic regurgitation rates than self-expanding Evolut valves but exhibit a higher incidence of PPM in S3 [36].

In a study by Fukui et al. in 2023, a CT-based analysis was employed to assess the feasibility of ViV procedures with the Sapiens 3 and Evolut valves [2]. This study examined how under-expanded S3 TAVR influenced the sizing decisions for subsequent TAVR replacements. They found that 92.1% of patients had under-expanded initial S3 TAVRs, necessitating deviations from standard in vitro sizing recommendations for the second TAVR in about 17% of cases. This deviation was primarily influenced by the expansion area of the initial TAVR, with a threshold below 89% leading to the selection of a smaller size than in vitro recommendations. Despite these variations, approximately 57-60% of patients undergoing S3-in-S3 and Evolut-in-S3 procedures were considered low risk for coronary complications. Key factors in reducing coronary risk included deep S3 implantation and the choice of Evolut for the second TAV, with the latter showing a notable 11% risk reduction in higher-risk patients. The study also highlighted a substantial difference in the risk of PPM between S3-in-S3 (21%) and Evolut-in-S3 (1%) procedures. The study indicates that redo-TAVR is generally feasible with a low risk to coronaries in about 60% of patients, influenced by various factors, including sizing strategy, TAVR type, native annular anatomy, and implant depth [2].

## Conclusions

In conclusion, this case involving a 75-year-old male patient who underwent ViV TAVR for severe prosthetic AVS, stemming from early SVD secondary to PPM in the context of severe obesity, underscores the vital importance of precise valve sizing in TAVR procedures. Despite the initial procedural success, the patient's rapid clinical decline post-TAVR highlights the challenges in valve selection and sizing, particularly considering individual factors such as BMI and BSA. This case necessitated a strategic reevaluation and subsequent ViV TAVR intervention to address PPM and improve hemodynamic outcomes. This underscores the critical role of ViV strategies in managing PPM-related complications, especially in obese patients. The case reinforces the necessity for thorough post-TAVR hemodynamic assessments using both flow-dependent and flow-independent metrics. It also illustrates the benefits of ViV procedures in minimizing operative risks and complications associated with primary TAVR valve failures. Moreover, this case illuminates potential research directions in enhancing valve sizing and selection strategies for obese patients, evaluating the benefits and risks of valve oversizing, and advancing prosthesis design to reduce PPM incidence. These considerations are crucial for optimizing patient outcomes and hemodynamic efficiency in TAVR.
